# Identification of the diagnostic genes and immune cell infiltration characteristics of gastric cancer using bioinformatics analysis and machine learning

**DOI:** 10.3389/fgene.2022.1067524

**Published:** 2023-01-04

**Authors:** Rongjun Xie, Longfei Liu, Xianzhou Lu, Chengjian He, Guoxin Li

**Affiliations:** ^1^ Department of General Surgery, Nanfang Hospital, The First School of Clinical Medicine, Southern Medical University, Guangzhou, China; ^2^ Department of General Surgery, Affiliated Nanhua Hospital, Hengyang Medical School, University of South China, Hengyang, China; ^3^ Guangdong Provincial Key Laboratory of Precision Medicine for Gastrointestinal Tumor, Nanfang Hospital, The First School of Clinical Medicine, Southern Medical University, Guangzhou, China; ^4^ Department of Intensive Care Medicine, Affiliated Nanhua Hospital, Hengyang Medical School, University of South China, Hengyang, China

**Keywords:** gastric cancer, diagnostic gene, immune cell infiltration, bioinformatics analysis, machine learning, LASSO, SVM-RFE

## Abstract

**Background:** Finding reliable diagnostic markers for gastric cancer (GC) is important. This work uses machine learning (ML) to identify GC diagnostic genes and investigate their connection with immune cell infiltration.

**Methods:** We downloaded eight GC-related datasets from GEO, TCGA, and GTEx. GSE13911, GSE15459, GSE19826, GSE54129, and GSE79973 were used as the training set, GSE66229 as the validation set A, and TCGA & GTEx as the validation set B. First, the training set screened differentially expressed genes (DEGs), and gene ontology (GO), kyoto encyclopedia of genes and genomes (KEGG), disease Ontology (DO), and gene set enrichment analysis (GSEA) analyses were performed. Then, the candidate diagnostic genes were screened by LASSO and SVM-RFE algorithms, and receiver operating characteristic (ROC) curves evaluated the diagnostic efficacy. Then, the infiltration characteristics of immune cells in GC samples were analyzed by CIBERSORT, and correlation analysis was performed. Finally, mutation and survival analyses were performed for diagnostic genes.

**Results:** We found 207 up-regulated genes and 349 down-regulated genes among 556 DEGs. gene ontology analysis significantly enriched 413 functional annotations, including 310 biological processes, 23 cellular components, and 80 molecular functions. Six of these biological processes are closely related to immunity. KEGG analysis significantly enriched 11 signaling pathways. 244 diseases were closely related to Ontology analysis. Multiple entries of the gene set enrichment analysis analysis were closely related to immunity. Machine learning screened eight candidate diagnostic genes and further validated them to identify *ABCA8*, *COL4A1*, *FAP*, *LY6E*, *MAMDC2*, and *TMEM100* as diagnostic genes. Six diagnostic genes were mutated to some extent in GC. *ABCA8*, *COL4A1*, *LY6E*, *MAMDC2*, *TMEM100* had prognostic value.

**Conclusion:** We screened six diagnostic genes for gastric cancer through bioinformatic analysis and machine learning, which are intimately related to immune cell infiltration and have a definite prognostic value.

## 1 Introduction

Gastric cancer (GC) is one of the most prevalent digestive system malignancies and the third leading cause of cancer-related deaths worldwide (Bray et al., 2018). Although surgery and chemotherapy have improved survival rates for advanced GC, the overall survival (OS) rate is still < 40%, and more than half of patients experience postoperative recurrence (Liu et al., 2016; Jiang et al., 2017). Due to the atypical early symptoms of GC, it is easy to overlook the fact that many patients are already in an advanced or even a terminal stage by the time they are diagnosed (Cao et al., 2021). Therefore, identifying novel and feasible biomarkers is essential for the early diagnosis and treatment of GC.

While immunotherapy has made significant breakthroughs in a variety of solid tumors, it has also provided new strategies and hope for the comprehensive treatment of GC (Robert et al., 2015; Zhou et al., 2020; Janjigian et al., 2021; Pietrantonio et al., 2021; Umeda et al., 2021). Unfortunately, not all GC patients can benefit from it (Chen and Mellman, 2017). Therefore, there is an urgent need for research into how to choose GC patients who will respond well to immunotherapy, predict its effectiveness, and get “inactive” GC patients to respond to and benefit from it.

The tumor microenvironment (TME) consists of a complex network of multiple types of stromal cells, immune cells, and extracellular components that surround tumor cells and are nourished by the vascular system (Turley et al., 2015). Studies have shown that the TME has a profound association with the efficacy of immunotherapy and that the profile of the TME significantly affects disease progression and regression (Diaz and Le, 2015; Hegde et al., 2016; Mariathasan et al., 2018; Hegde and Chen, 2020; Helmink et al., 2020). Therefore, systematically resolving the phenotypes of different cells in TME, especially the characteristics of immune cell infiltration, is key to understanding the genesis and progression of many tumors, including GC, and improving immunotherapy’s effectiveness. Based on linear support vector regression, CIBERSORT is an algorithm for the deconvolution of expression matrices of human immune cell subtypes. When comparing several methodologies, deconvolution analysis of expression matrices of unknown mixtures with similar cell types emerges as the most effective. The approach generates gene expression profiles for 22 distinct types of immune cells based on a previously established reference set (Newman et al., 2015). Using this method, we now have a solid foundation for investigating TME.

In recent years, machine learning (ML) has been widely used to solve various complex problems in the medical field (Shehab et al., 2022). It is capable of mining vast amounts of data and discovering exciting hidden relationships within them, providing explanations and defining patterns. It can help improve disease diagnosis accuracy, reliability, and predictability. With the continuous development of gene chips and high-throughput sequencing technologies, bioinformatics data have exploded in just a few decades. The joint application of bioinformatics analysis and ML is increasing and shows great potential (Kononenko, 2001). No study has seen the use of ML to identify and characterize GC-related diagnostic genes, which deserves further exploration.

This study analyzed the differentially expressed genes (DEGs) between GC (tumor) and normal gastric (normal) tissue samples through GC-related data in public databases (GEO, TCGA, and GTEx) to explore their biological functions and signaling pathways. We adopted a combined bioinformatics analysis and ML strategy to screen and validate the targeted genes associated with GC diagnosis using the LASSO (least absolute shrinkage and selection operator) and SVM-RFE (support vector machine recursive feature elimination) algorithms. The infiltration of each immune cell in GC was then analyzed using CIBERSORT. Finally, we performed a correlation analysis between diagnostic genes and immune cell infiltration characteristics to gain insight into the molecular immune mechanisms involved in developing GC.

## 2 Materials and methods

### 2.1 Data collection and processing

We retrieved and downloaded six GC datasets from the GEO database: GSE13911, GSE15459, GSE19826, GSE54129, GSE66229, and GSE79973. Their data are based on the Affymetrix Human Genome U133 Plus 2.0 Array of GPL570 platform. The probe matrices were converted to gene matrices and normalized. GSE13911, GSE15459, GSE19826, GSE54129, and GSE79973, which contained 371 GC and 77 normal gastric tissue samples, were merged to remove batch effects (Johnson et al., 2007; Taminau et al., 2012) and used as the training set (Supplementary Figure S1A–E). The GSE66229 dataset, which included 300 GC samples and 100 paired normal gastric tissue samples, was used as validation set A. We downloaded RNA sequencing data (FPKM) of GC with mutation data from the TCGA database and downloaded RNA sequencing data (FPKM) of normal gastric tissues from the GTEx database. We combined the RNA sequencing data from the TCGA and GTEx databases to remove the batch effect as validation set B. In total, 375 GC samples were collected, including 207 normal gastric tissue samples.

### 2.2 Differential expression analysis

The R package “limma” was used to filter the DEGs between the tumor and normal groups in the training set. The filtering conditions were set to |logFC| > 1 and FDR < 0.05.

### 2.3 Functional enrichment analysis

Gene ontology (GO), kyoto encyclopedia of genes and genomes (KEGG), and disease ontology (DO) analyses were performed using the R packages “clusterProfiler,” “org.Hs.eg.db,” “DOSE,” and “enrichplot” to observe the enrichment of the DEGs in function, pathway, and disease. Gene set enrichment analysis (GSEA) was performed using the R package “GSEABase” to observe functional and pathway differences between the tumor and normal groups; q < 0.05 was considered statistically significant.

### 2.4 Screening and validation of diagnostic genes

The LASSO and SVM-RFE ML algorithms were used to screen candidate diagnostic genes. LASSO is a compressive estimation method that creates a more accurate model by building a penalty function that forces it to compress some regression coefficients or force the sum of the absolute values of the coefficients to be less than a fixed value while setting some coefficients to zero. Thus, it retains the benefit of subset shrinkage and is a biased estimator when dealing with complex covariance in data. SVM-RFE is a sequential backward selection algorithm that uses the support vector machine principle of the maximum interval. It trains the sample with the model and then ranks each feature by its score, removes the feature with the lowest score, trains the model again with the remaining features for the next iteration, and chooses the required number of features. It is an embedded-based method that improves learning performance by using the principle of minimizing structural risk while minimizing empirical error. The R package “glmnet” for LASSO and the R package “e1071” for SVM-RFE were used. The intersection of the results of the two algorithms is the candidate diagnostic gene. Receiver operating characteristic (*R*OC) curves assessed the predictive effect of candidate diagnostic genes in the training and validation sets. The differential expression of candidate diagnostic genes between the tumor and normal groups was also analyzed; *p* < 0.05 was considered to be statistically significant.

### 2.5 Immune cell infiltration analysis

The differences in immune cell infiltration between the tumor and normal groups were analyzed using the R package “CIBERSORT” to obtain the infiltration of 22 immune cells in each training set sample. p < 0.05 was considered statistically significant. Furthermore, the correlation between diagnostic genes and immune infiltrating cells was calculated; |*R*| ≥ 0.3 and *p* < 0.05, considered to be correlated.

### 2.6 Analysis of diagnostic gene mutations

We investigated the mutation status of the diagnostic genes using the R package “maftools” and the TCGA database’s GC mutation data.

### 2.7 Survival analysis of diagnostic genes

Online survival analysis was performed using the GC data from the Kaplan Meier Plotter database to select the optimal cut-off value calculated by the system. We evaluated the effects of diagnostic genes on the OS, first progression (FP), and post-progression survival (PPS) of GC patients; *p* < 0.05 was considered to be statistically significant.

## 3 Results

### 3.1 Screening for DEGs

**FIGURE 1:**
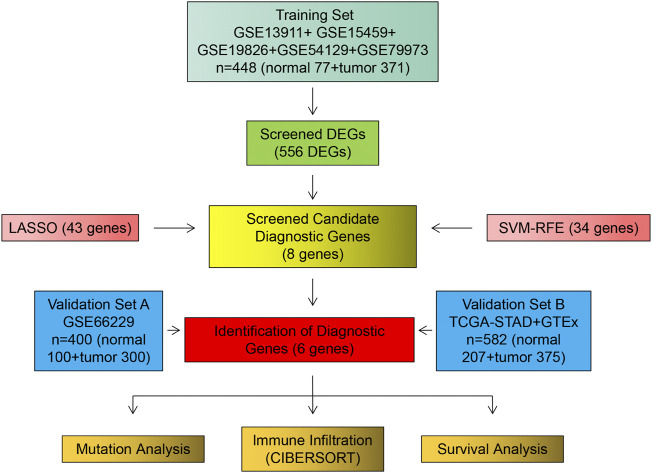
Flow chart of research design and analysis.

**FIGURE 2:**
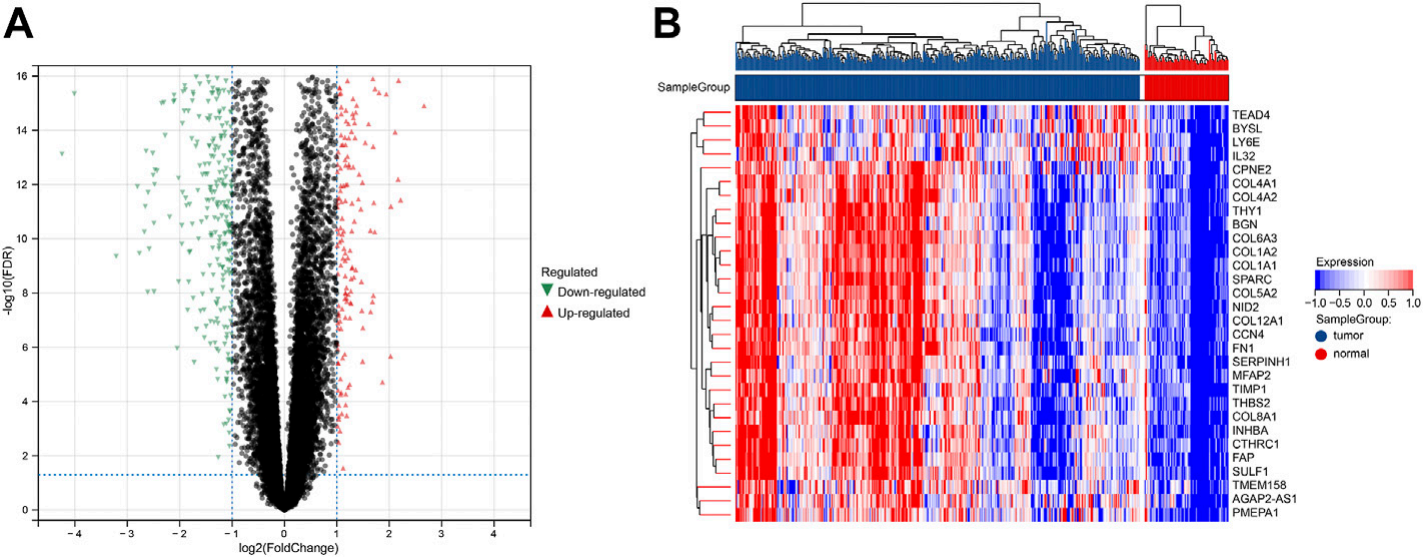
DEGs between tumor and normal groups in the training set. **(A)** Volcano plot of DEGs with difference folds >2, red for up-regulated and green for down-regulated. **(B)** Heat map of DEGs, red represents high expression, and blue represents low expression.

The procedure followed during this research is shown in Figure 1. A total of 556 eligible DEGs were screened from the training set according to the previously described filtering conditions, with 207 up-regulated and 349 down-regulated in the tumor group (Supplementary Table S1 and Figures 2A, B).

### 3.2 Functional enrichment analysis

**FIGURE 3:**
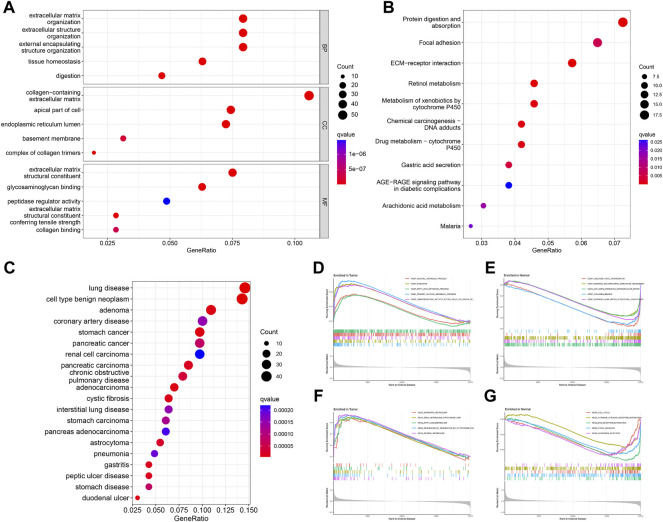
Enrichment analysis of DEGs. GO **(A)**, KEGG **(B)**, and DO **(C)** analysis of the enrichment of DEGs for function, pathways, and disease, and GSEA analysis of differences in function **(D,E)** and pathways **(F,G)** between the tumor and normal groups.

GO analysis showed that DEGs were significantly enriched for 413 functional annotations, including 310 biological processes, 23 cellular components, and 80 molecular functions (Supplementary Table S2 and Figure 3A). The significantly enriched biological processes related to immunity included the following: the antimicrobial humoral immune response mediated by antimicrobial peptide, the humoral immune response, the organ or tissue specific immune response, the mucosal immune response, the innate immune response in mucosa, and the mature B cell differentiation involved in immune response. KEGG analysis indicated that DEGs significantly enriched 11 signaling pathways (Supplementary Table S3 and Figure 3B). DO analysis revealed that the DEGs were closely associated with 244 diseases (Supplementary Table S4 and Figure 3C). The results of the GSEA analysis demonstrated differences in function and pathways between the tumor and normal groups, with multiple entries closely associated with immunity (Supplementary Tables S5, 6 and Figures 3D–G).

### 3.3 Screening and validation of diagnostic genes

**FIGURE 4:**
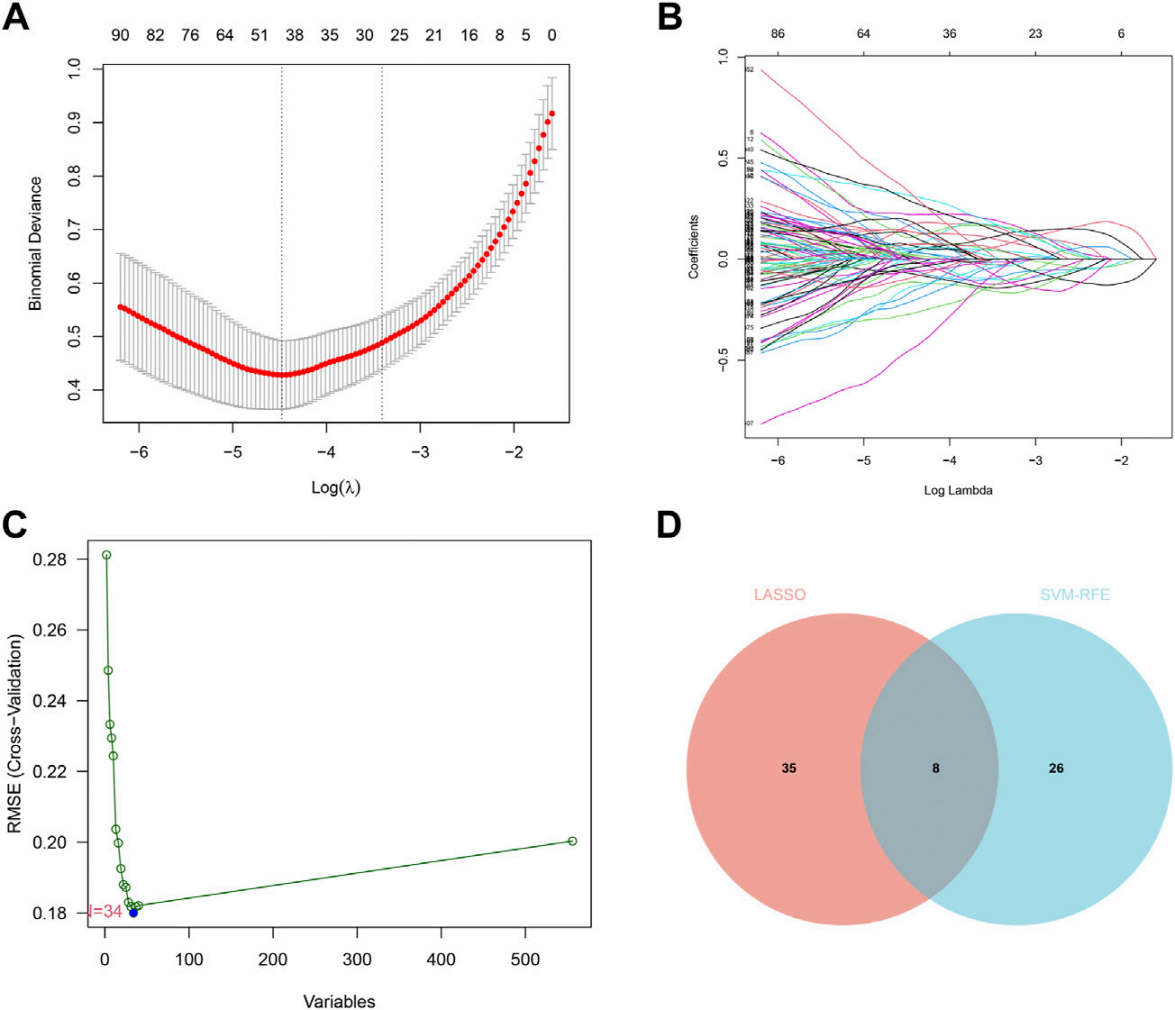
LASSO and SVM-RFE screening of candidate diagnostic genes.**(A)** LASSO screening of candidate diagnostic genes, with logλ on the horizontal axis and cross-validation error on the vertical axis. The cross-validation error is minimal when 43 genes are selected. **(B)** Different colored lines represent different genes screened by LASSO. **(C)** SVM-RFE screening of candidate diagnostic genes. The horizontal axis represents the change in the number of genes, and the vertical axis represents the cross-validation error. The cross-validation error was minimized when *n* = 34. **(D)** The Venn diagram displays the intersection of the results of the two algorithms.

**FIGURE 5:**
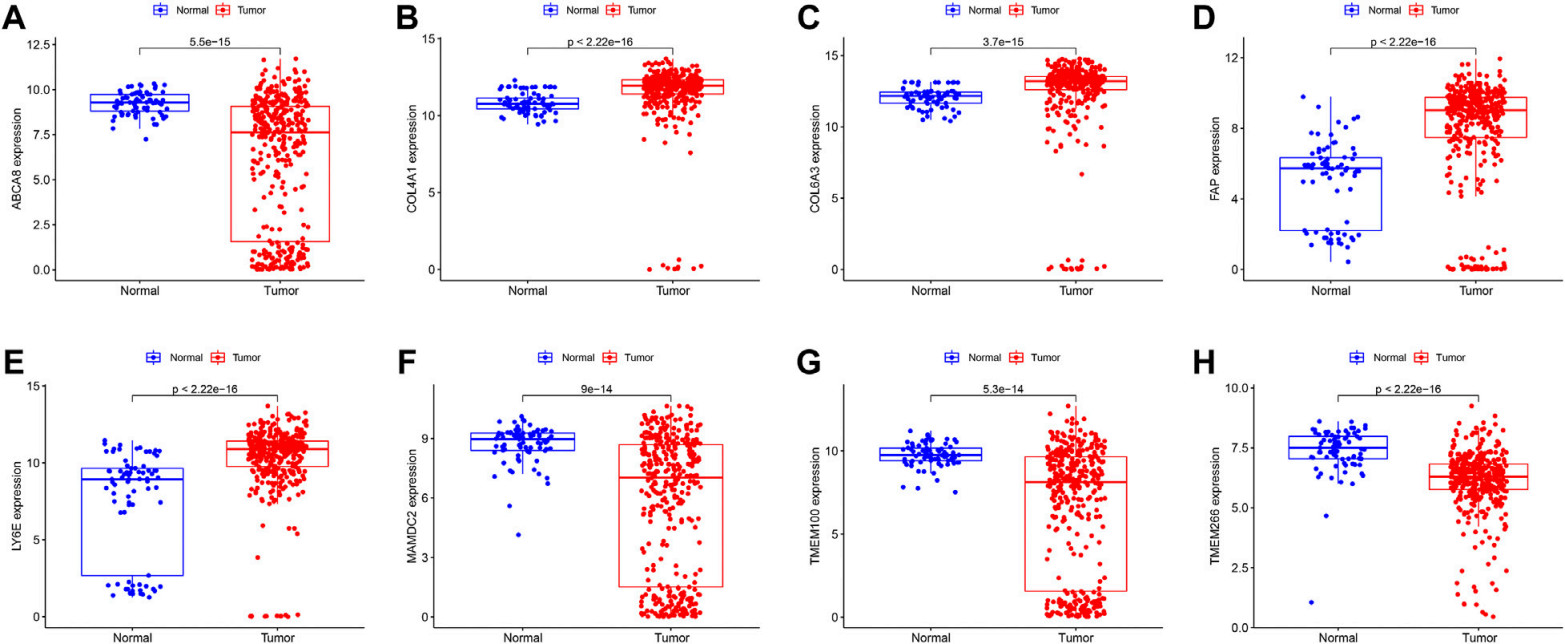
Expression of candidate diagnostic genes in the training set **(A–H)** Scatter plots showing the expression of candidate diagnostic genes between tumor and normal groups in the training set. Red indicates the tumor group and blue indicates the normal group. p < 0.05 indicates significant difference.

**FIGURE 6:**
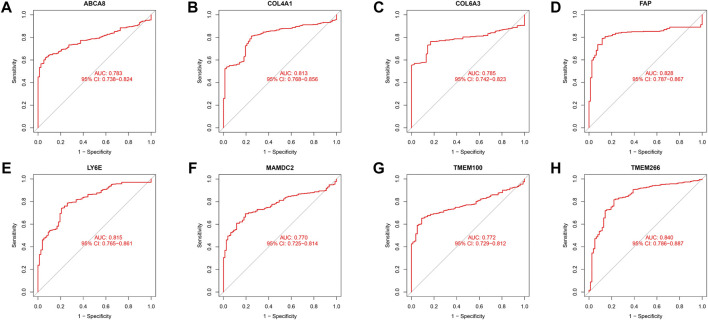
ROC curves in the training set. **(A–H)** The ROC curves for the eight candidate diagnostic genes in the training set are shown in the figure. The horizontal coordinate is the false positive rate, presented as 1-specificity, and the vertical coordinate is the true positive rate, presented as sensitivity.

We identified 43 diagnosis-associated genes using the LASSO algorithm (Figures 4A, B) and 34 using the SVM-RFE algorithm (Figure 4C) from DEGs. The overlapping genes of both algorithms were *ABCA8*, *COL4A1*, *COL6A3*, *FAP*, *LY6E*, *MAMDC2*, *TMEM100*, and *TMEM266* as candidate diagnostic genes (Figure 4D). *COL4A1*, *COL6A3*, *FAP*, and *LY6E* were up-regulated in the tumor group in the training set, whereas *ABCA8*, *MAMDC2*, *TMEM100*, and *TMEM266* were down-regulated (Figures 5A–H). All eight genes showed alterations consistent with the training set in validation sets A and B. Only *COL6A3* showed no statistically significant difference in validation set A (Supplementary Figures S2A–H and Figures 3A–H). The area under the curve (AUC) values of *ABCA8*, *COL4A1*, *COL6A3*, *FAP*, *LY6E*, *MAMDC2*, *TMEM100*, and *TMEM266* in the training set are 0.783, 0.813, 0.785, 0.828, 0.815, 0.770, 0.772, and 0.840, respectively, all of which are greater than 0.70, showing a higher precision predictive value (Figures 6A–H). The AUC values in validation set A were 0.950, 0.790, 0.552, 0.830, 0.887, 0.967, 0.957, and 0.634, all of which were greater than 0.70 except for *COL6A3* and *TMEM266* (Supplementary Figures S4A–H). Meanwhile, the AUC values in validation set B were 0.945, 0.895, 0.680, 0.707, 0.943, 0.910, 0.931, and 0.800, which were greater than 0.70 except for *COL6A3* (Supplementary Figures S5A–H). *ABCA8*, *COL4A1*, *FAP*, *LY6E*, *MAMDC2*, and *TMEM100* showed consistent AUC values in the training and validation sets, and all of them were greater than 0.70, with high prediction accuracy and reliability and repeatability. Therefore, we identified them as diagnostic genes.

### 3.4 Immune cell infiltration analysis

**FIGURE 7:**
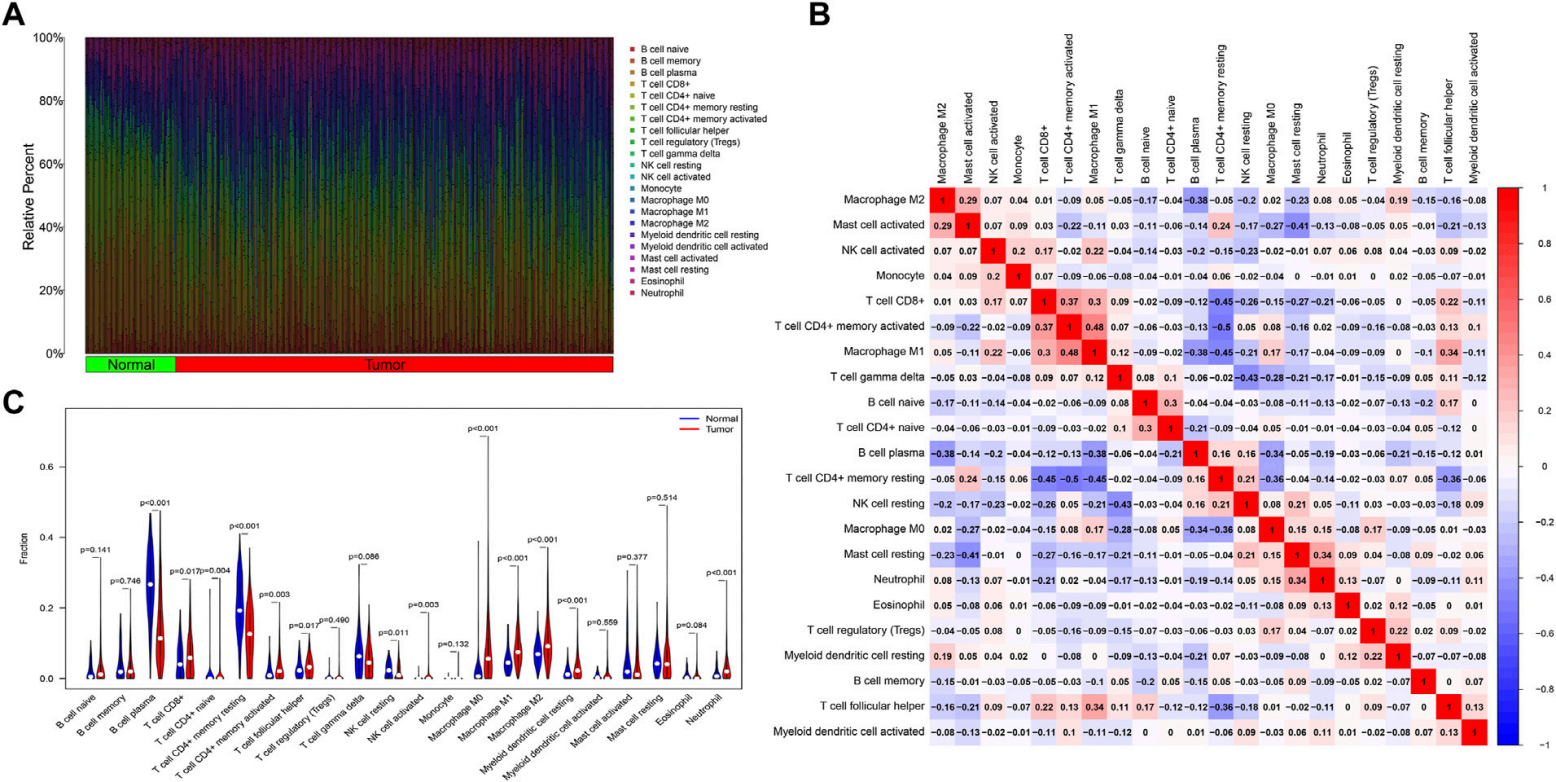
Analysis of immune cell infiltration. **(A)** The graph shows the degree of infiltration of different immune cells between the tumor and normal groups. **(B)** Immune cell correlation analysis. The horizontal and vertical axes are the names of immune cells, and the values indicate the correlation coefficients between immune cells. The red color indicates a positive correlation, and the blue indicates a negative one. **(C)** Violin plot showing the difference of immune infiltrating cells between tumor and normal groups. The horizontal axis indicates the name of immune cells, and the vertical axis indicates the content of immune cells. Blue indicates the normal group, and red indicates the tumor group. *p* < 0.05 indicates a significant difference.

We obtained the proportion of 22 immune cell infiltrations in each sample of the training set using the CIBERSORT algorithm (Figure 7A). The correlation heat map between each immune cell demonstrated (Supplementary Table S7 and Figure 7B) that Macrophage M1 was positively correlated with T cell CD4^+^ memory activated (*R* = 0.48, *p* < 0.05), T cell follicular helper (*R* = 0.34, *p* < 0.05), and T cell CD8^+^ (*R* = 0.30, *p* < 0.05). T cell CD4^+^ memory activated was positively correlated with T cell CD8^+^ (*R* = 0.37, *p* < 0.05). Neutrophil was positively correlated with Mast cell resting (*R* = 0.34, *p* < 0.05). T cell CD4^+^ naive was positively correlated with B cell naive (*R* = 0.30, *p* < 0.05). Conversely, T cell CD4^+^ memory resting was negatively correlated with T cell CD4^+^ memory activated (*R* = −0.50, *p* < 0.05), Macrophage M1 (*R* = −0.45, *p* < 0.05), T cell CD8^+^ (*R* = −0.45, *p* < 0.05), Macrophage M0 (*R* = −0.36, *p* < 0.05), and T cell follicular helper (*R* = −0.36, *p* < 0.05). B cell plasma had a negative correlation with macrophage M1 (*R* = −0.38, *p* < 0.05), M2 (*R* = −0.38, *p* < 0.05), and M0 (*R* = −0.34, *p* < 0.05). T cell gamma delta was negatively correlated with NK cell resting (*R* = −0.43, *p* < 0.05). Mast cell activated was negatively correlated with Mast cell resting (*R* = −0.41, *p* < 0.05).

Further analysis revealed a significant difference in the proportion of the infiltration of 13 immune cell types between the tumor and normal groups (Supplementary Table S8 and Figure 7C). In the tumor group, T cell CD8^+^, T cell CD4^+^ naive, T cell CD4^+^ memory activated, T cell follicular helper, NK cell activated, Macrophage M0, Macrophage M1, Macrophage M2, Myeloid dendritic cell resting, and Neutrophil infiltration were higher in proportion (*p* < 0.05). The proportion of B cell plasma, T cell CD4^+^ memory resting, and NK cell resting infiltration was higher in the normal group (*p* < 0.05). The findings above revealed substantial differences in the characteristics of immune cell infiltration between tumor and normal tissues and a complex interrelationship between the various immune cells infiltrating in TME.

### 3.5 Correlation analysis of diagnostic genes with infiltrating immune cells

**FIGURE 8:**
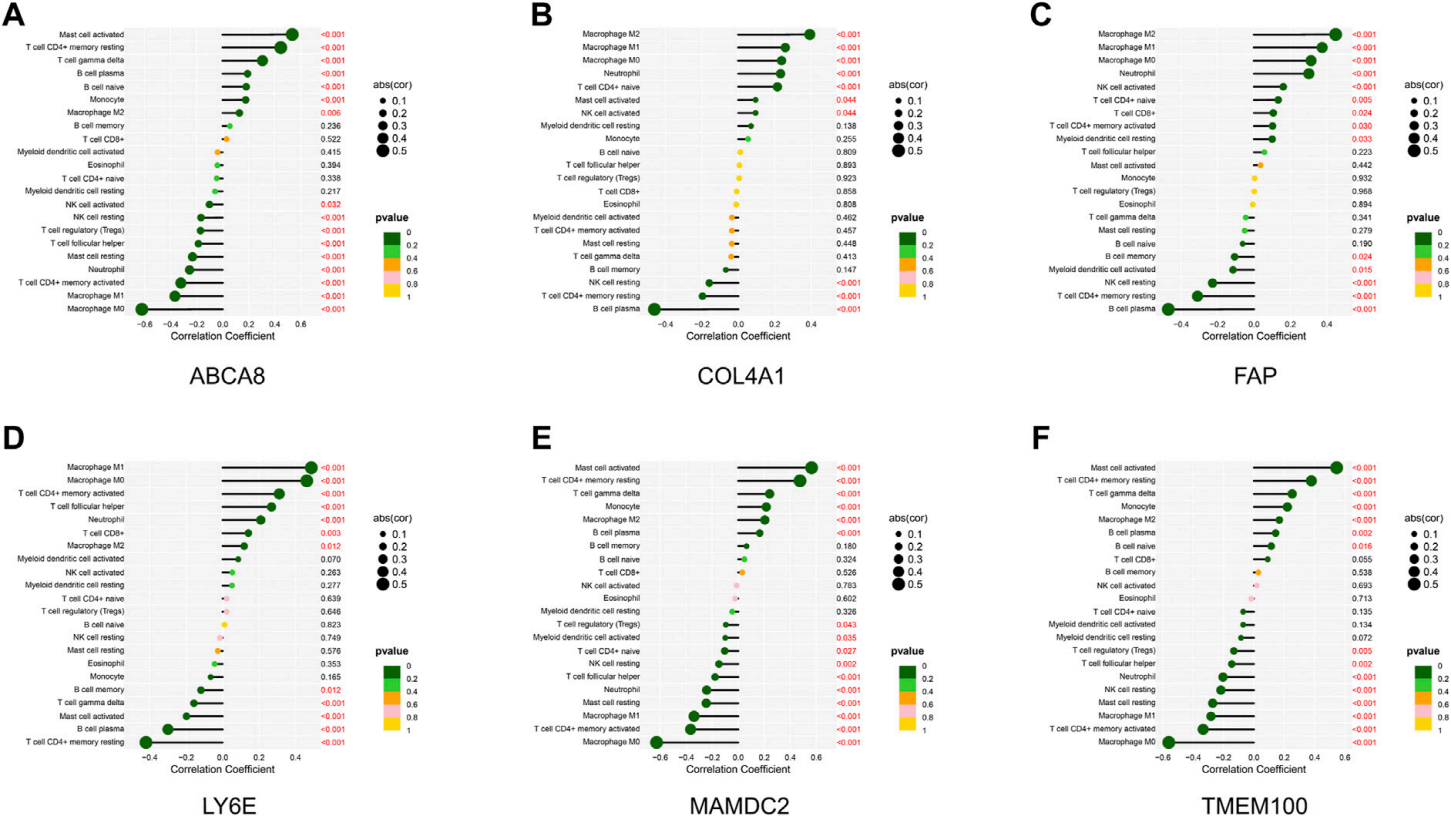
Correlation analysis of diagnostic genes and immune infiltrating cells. **(A–F)** Correlation between diagnostic genes and immune infiltrating cells. Horizontal coordinates indicate correlation coefficients, and vertical coordinates indicate immune cell names. The circle size means the absolute value of the correlation coefficient, the color indicates the *p*-value of the correlation test, and the *p*-value size is indicated by color.

Through the correlation analysis of diagnostic genes and infiltrating immune cells (Supplementary Table S9 and Supplementary Figures S6A–F), we discovered that *ABCA8* was significantly positively correlated with T cell gamma delta (*R* = 0.31, *p* < 0.05), T cell CD4^+^ memory resting (*R* = 0.45, *p* < 0.05), and Mast cell activated (*R* = 0.54, *p* < 0.05) and negatively correlated with Macrophage M0 (*R* = −0.62, *p* < 0.05), Macrophage M1 (*R* = −0.37, *p* < 0.05), and T cell CD4^+^ memory activated (*R* = −0.32, *p* < 0.05) (Figure 8A). *COL4A1* was significantly positively correlated with Macrophage M2 (*R* = 0.39, *p* < 0.05) and negatively correlated with B cell plasma (*R* = −0.46, *p* < 0.05) (Figure 8B). *FAP* was significantly positively correlated with Neutrophil (*R* = 0.30, *p* < 0.05), Macrophage M0 (*R* = 0.31, *p* < 0.05), Macrophage M1 (*R* = 0.38, *p* < 0.05), and Macrophage M2 (*R* = 0.45, *p* < 0.05) and negatively correlated with B cell plasma (*R* = −0.47, *p* < 0.05) and T cell CD4^+^ memory resting (*R* = −0.31, *p* < 0.05) (Figure 8C). *LY6E* was significantly positively correlated with T cell CD4^+^ memory activated (*R* = 0.31, *p* < 0.05), Macrophage M0 (*R* = 0.46, *p* < 0.05), and Macrophage M1 (*R* = 0.49, *p* < 0.05) and negatively correlated with T cell CD4^+^ memory resting (*R* = −0.42, *p* < 0.05) and B cell plasma (*R* = −0.30, *p* < 0.05) (Figure 8D). *MAMDC2* was significantly positively correlated with T cell CD4^+^ memory resting (*R* = 0.47, *p* < 0.05) and Mast cell activated (*R* = 0.56, *p* < 0.05) and negatively correlated with Macrophage M0 (*R* = −0.63, *p* < 0.05), T cell CD4^+^ memory activated (*R* = −0.37, *p* < 0.05), and Macrophage M1 (*R* = −0.34, *p* < 0.05) (Figure 8E). *TMEM100* was significantly positively correlated with T cell CD4^+^ memory resting (*R* = 0.38, *p* < 0.05) and Mast cell activated (*R* = 0.55, *p* < 0.05) and negatively correlated with Macrophage M0 (*R* = −0.56, *p* < 0.05) and T cell CD4^+^ memory activated (*R* = −0.33, *p* < 0.05) (Figure 8F). The results above imply an intimate and comprehensive association between diagnostic genes and immune infiltrating cells, which interact with each other to influence the immune infiltration characteristics of TME.

### 3.6 Analysis of diagnostic gene mutations

**FIGURE 9:**
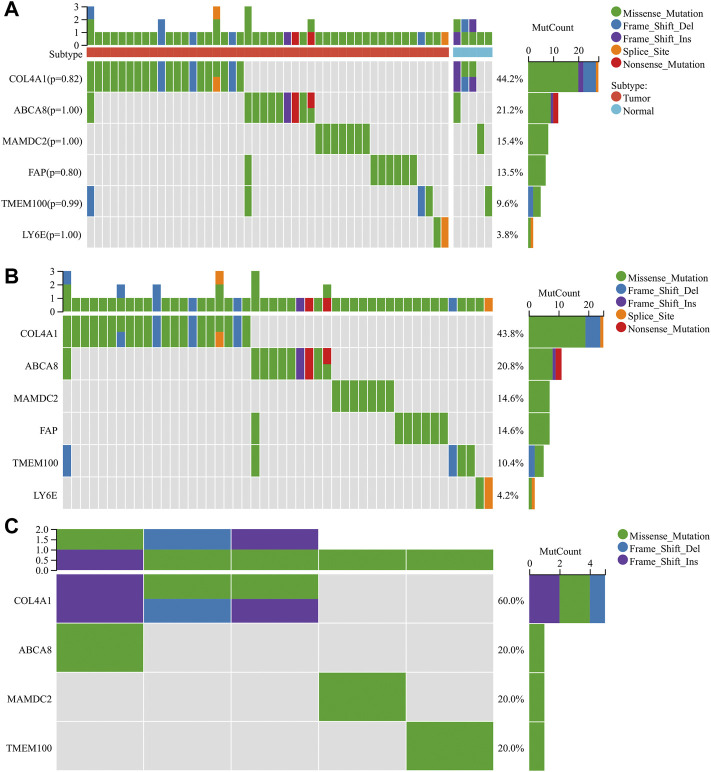
Mutation analysis of diagnostic genes **(A)** Mutations of diagnostic genes between tumor and normal groups. **(B)** Mutations of diagnostic genes in the tumor group. **(C)** Mutations of diagnostic genes in the normal group.

We performed the mutation analysis of diagnostic genes using GC mutation data from the TCGA database. The results revealed that, in descending order, the most common mutation types in the tumor group were Missense_Mutation, Frame_Shift_Del, Frame_Shift_Ins, Splice_Site, and Non-sense_Mutation (Figure 9B). The mutation types in the normal group were Missense_Mutation, Frame_Shift_Del, and Frame_Shift_Ins (Figure 9C). Six diagnostic genes were mutated in descending frequency in the tumor group: *COL4A1*, *ABCA8*, *MAMDC2*, *FAP*, *TMEM100,* and *LY6E* (Figure 9B). Four diagnostic genes were mutated in descending frequency in the normal group: *COL4A1*, *ABCA8*, *MAMDC2*, and *TMEM100* (Figure 9C). The *COL4A1*, *ABCA8*, *MAMDC2*, *FAP*, *TMEM100*, and *LY6E* mutation frequencies did not differ significantly between the tumor and normal groups (*p* > 0.05) (Figure 9A).

### 3.7 Survival analysis of diagnostic genes

**FIGURE 10:**
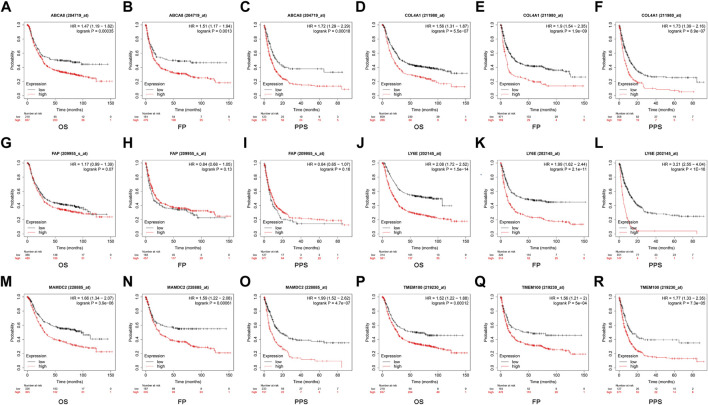
Survival analysis of candidate diagnostic genes **(A–R)** Effect of each diagnostic gene on overall survival (OS), first progression (FP), and post-progression survival (PPS).

The optimal cut-off values calculated by the system were selected using the GC data from the Kaplan Meier Plotter database for online survival analysis. The results indicated that *ABCA8* (Figures 10A–C), *COL4A1* (Figures 10D–F), *LY6E* (Figures 10J–L), *MAMDC2* (Figure 10M–O), and *TMEM100* (Figures 10P–R) effectively predicted OS, FP, and PPS (*p* < 0.05) in GC patients, while *FAP* could not predict OS, FP, and PPS (*p* > 0.05) (Figures 10G–I).

## 4 Discussion

GC has a high incidence and mortality rate, and the prognosis is closely related to the timing of diagnosis and treatment. The 5-year survival rate of early-stage patients is over 90%, while that for those at an advanced stage is less than 20% (Liu et al., 2016; Jiang et al., 2017; Bray et al., 2018; Cao et al., 2021). A timely diagnosis and treatment can increase the survival rate and reduce mortality. Immunotherapy breakthroughs have given insights into GC (Robert et al., 2015; Zhou et al., 2020; Janjigian et al., 2021; Pietrantonio et al., 2021; Umeda et al., 2021), and immune cell infiltration characteristics are closely related to treatment outcomes (Diaz Jr and Le, 2015; Turley et al., 2015; Hegde et al., 2016; Chen and Mellman, 2017; Mariathasan et al., 2018; Hegde and Chen, 2020; Helmink et al., 2020). ML, which can extract relevant information from large amounts of data and uncover important associations, is increasingly used in the biomedical field (Kononenko, 2001; Shehab et al., 2022). In this study, we screened the DEGs of GC by bioinformatics analysis and performed functional and pathway profiling. Then, eight candidate diagnostic genes were filtered from the DEGs using the LASSO and SVM-RFE algorithms, and their diagnostic efficacy and differential expression were cross-checked in the training and validation sets. Next, the infiltration of each immune cell in GC was analyzed, and we assessed the correlation between diagnostic genes and immune cell infiltration characteristics. Finally, the diagnostic genes were analyzed for mutation and survival.

The GO analysis of DEGs significantly enriched six biological processes related to immunity. The KEGG analysis significantly enriched 11 signaling pathways related to intercellular communication, tumorigenesis, and metabolism, including ECM-receptor interaction, chemical carcinogenesis, and various metabolic processes. The DO analysis significantly enriched 244 diseases, including GC and other malignancies. The GSEA analysis between the tumor and normal samples was also enriched for several immune and tumor-related entries. Accordingly, our findings imply that DEGs are closely related to immunity and tumors.

We used 2 ML algorithms to screen the candidate diagnostic genes of GC from DEGs. LASSO can achieve the selection of variables while estimating parameters, thus better solving the problem of multicollinearity in regression analysis and better explaining the results (Rafique et al., 2021). Additionally, SVM-RFE is different from other statistical methods because it does not follow the traditional path from induction to deduction. Instead, it uses efficient transductive inference and makes problems such as classification and regression much easier to solve (Tang et al., 2020). After taking the intersection of the results of the two algorithms, we obtained the candidate diagnostic genes. The results of the cross-test between the training and validation sets revealed that the differential expression of the candidate diagnostic genes showed consistent changes, among which the AUC values of *ABCA8*, *COL4A1*, *FAP*, *LY6E*, *MAMDC2*, and *TMEM100* in both the training and validation sets were greater than 0.7, which had higher diagnostic efficacy and stability. Therefore, we identified them as diagnostic genes.

The extracellular matrix (ECM) has an abundance of collagen, which plays an important role in regulating TME and tumor cell behavior (Järveläinen et al., 2009; Dong et al., 2014). Collagen IV is the most abundant component of the ECM basement membrane (Kalluri, 2003). *COL4A1* (Collagen Type IV Alpha 1 Chain), a collagen IV molecule, has two distinct integrin α1β1 and α2β1 recognition sites (Kühn, 1995) and is involved in intercellular interactions. Cui X et al. found that *COL4A1* expression was elevated in GC tissues and cells and that the knockdown of its expression inhibited cell proliferation, migration, invasion, and EMT in GC. The specific mechanism was that the downregulation of *COL4A1* suppressed the aggressive phenotype of GC cells by blocking the Hedgehog signaling pathway (Cui et al., 2022). Cancer-associated fibroblasts (CAFs) are an important component of TME and play an important role in tumor invasion and metastasis (Czekay et al., 2022). *FAP* (Fibroblast Activation Protein Alpha) is a specific marker of CAFs and belongs to the serine protease family of type II integral membrane glycoproteins (Yang et al., 2016). Wang RF et al. found that *FAP* was overexpressed in the CAFs of GC tissues and that the expression level of *FAP* in CAFs was significantly correlated with Lauren’s classification, grade of differentiation, depth of tumor infiltration, and TNM stage but not with patient age and gender. When MGC-803 GC cells were co-cultured with CAFs, the invasive and migratory ability of the MGC-803 cells was also significantly increased. In contrast, the invasive and migratory abilities of the MGC-803 cells decreased considerably after knocking down *FAP* in CAFs. Hence, *FAP* may be an important regulator of GC invasion and migration (Wang et al., 2013). *LY6E* (Lymphocyte Antigen 6 Family Member E) encodes a GPI-anchored cell surface protein that regulates T lymphocytes’ proliferation, differentiation, and activation (Upadhyay, 2019). In Lv et al. (2018)’s study, *LY6E* expression was elevated in GC tissues and cells, and it was associated with histological grading, AJCC staging, and tumor location in GC. The knockdown of *LY6E* by targeted siRNA could inhibit the growth, proliferation, and migration of GC cell. *TMEM100* (transmembrane protein 100) encoded products play an important role in embryonic arterial endothelial cell differentiation and vascular morphogenesis (Zhuang et al., 2020). Zhuang J et al. found that *TMEM100* expression was significantly downregulated in GC samples. The overexpression of *TMEM100* inhibited the migration and invasion of GC cells but did not affect their growth. The down-regulation of *TMEM100* restored the migratory and invasive ability of GC cells. Moreover, the upregulation of *TMEM100* increased the sensitivity of GC cells to chemotherapeutic drugs such as 5-fluorouracil and cisplatin. As a result, the authors reasoned that *TMEM100*, a GC inhibitory factor, may be a therapeutic target and prognostic indicator (Zheng et al., 2022).

*ABCA8* (ATP Binding Cassette Subfamily A Member 8) is a transmembrane transporter responsible for transporting organic compounds (e.g., cholesterol) and drug efflux (Sasaki et al., 2018). It belongs to the ATP-binding cassette (ABC) transporter superfamily. The ABC transporter-mediated anticancer drug efflux is a common mechanism of chemoresistance (Trigueros-Motos et al., 2017), and Yang C et al. discovered that *ABCA8* expression was significantly increased in human pancreatic cancer (PC) cells after gemcitabine (GEM) treatment and in GEM-resistant (Gem-R) PC cells. The knockdown of *ABCA8* reversed the chemo-resistant phenotype of Gem-R cells, whereas *ABCA8* overexpression significantly decreased the sensitivity of PC cells to GEM, suggesting an important role for *ABCA8* in regulating chemo-sensitivity (Yang et al., 2021). The MAM (meprin/A-5 protein/receptor protein-tyrosine phosphatase mu) structural domain is a conserved protein structural domain in various cell surface proteins (Kim et al., 2022). *MAMDC2* (MAM Domain Containing 2), a member of the MAM family, encodes a secretory protein consisting of 686 amino acids and contains a short N-terminal signal sequence and four contiguous MAM structural domains (Chin et al., 2005). Lee et al. (2020) observed that *MAMDC2* expression was down-regulated in breast cancer and that the overexpression of *MAMDC2* significantly inhibited the proliferation of breast cancer T-47D cells, which may act by attenuating the MAPK signaling pathway. Unfortunately, we did not find any experimental reports of *ABCA8* and *MAMDC2* associated with GC. In addition, since our results showed that *COL6A3* (Collagen Type VI Alpha 3 Chain) and *TMEM266* (Transmembrane Protein 266) had low AUC values in the validation set and poor diagnostic efficacy and stability, we will not elaborate on them here.

Immunotherapy has broken the previous monopoly of surgery, chemotherapy, and targeted therapy in GC treatment and has significantly improved the survival of some patients (Coleman et al., 2017; Kang et al., 2017; Shitara et al., 2018). Still, only 11–25% of GC patients can benefit from it (Chen and Mellman, 2017; Kang et al., 2017; Shitara et al., 2020). Therefore, it is a critical clinical issue to find biomarkers that can accurately predict the response to immunotherapy, discover the resistance mechanism of this therapy, and develop corresponding individualized treatment plans to avoid the harm and burden caused by the over- and inappropriate treatment of patients. Currently, the biomarkers used to predict the efficacy of PD-1/PD-L1 monoclonal antibodies include immunohistochemical expression levels of PD-L1 (CPS) (Kim et al., 2018), high microsatellite instability (MSI-H) (Diaz Jr and Le, 2015), and tumor mutational load (TMB) (Samstein et al., 2019). However, since these biomarkers all focus on intrinsic tumor characteristics with significant heterogeneity and neglect the assessment of the TME, the soil, and the ecosystem on which tumor growth depends, the stability of their predictive efficacy is limited.

Researchers have focused on the differences between tumor cells and normal cells in the past but neglected other non-tumor cells in tumor tissue. With the progress of research, the importance of TME on tumor development has been gradually recognized. Tumor cells are not separate entities; their microenvironment also affects carcinogenesis and development. Various types of immune cells and mesenchymal cells infiltrated in TME play an important role in tumor killing and immune escape (Turley et al., 2015). Wang JT et al. found that high IL17 mRNA expression and the high infiltration of IL17-positive cells within the tumor were associated with good prognosis in GC patients and that patients with high IL17-positive cell infiltration in GC tissue had a higher response rate to 5-FU-based postoperative adjuvant chemotherapy. A comparison of the analysis of TME-infiltrating immune cells, cytotoxic effector cytokines, and immune checkpoint molecules in patients from different IL17-expressing groups revealed that high IL17 mRNA expression and the high infiltration of IL17-positive cells in GC tissue were associated with more anti-tumor mast cell and NK cell infiltration and less pro-tumor M2 macrophage infiltration, while high IL17 mRNA expression was closely associated with an increased expression of anti-tumor cytokines, such as interferon-γ, perforin, granzyme A, and granzyme B. The results suggest that in the TME of GC patients, tumor-infiltrating IL17-positive cells promote anti-tumor immune responses by promoting the infiltration of anti-tumor immune effector cells and increasing the expression of anti-tumor immune effector molecules (Wang et al., 2019). Predina J et al. found that recurrent tumors were similar in size to primary tumors and that tumor cells were not phenotypically or functionally altered but were more resistant to drugs. The reason for this is the difference in immune infiltrating cells in the TME, with primary tumors having healthy anti-tumor effector CD8^+^ T cells. In contrast, recurrent tumors contain many immunosuppressive tumor-associated macrophages (TAMs) and Treg cells and the cytokines VEGF, IL-1β, IL-6, IL-10, and TGF-β, which suppress CD8^+^ T cells (Predina et al., 2013). Zeng D et al. investigated the relationship between immune cell infiltration and prognosis in the TME of GC patients and found that the infiltration levels of CD8^+^ T cells and M1 macrophages were significantly and positively correlated with prognosis. In contrast, the infiltration levels of M2 macrophages and resting CD4^+^ T cells were significantly correlated with poor prognosis. It was also found that the immune score established based on TME immune infiltrated cells greatly improved the accuracy of prognosis determination and was associated with the efficacy of chemotherapy (Zeng et al., 2018). Our results also show significant differences in the infiltrated immune cells between the tumor and normal groups and, more importantly, an intricate and inextricable association between the infiltrated immune cells and their diagnostic genes. Our research also further confirms that the characteristics of immune cell infiltration in TME are closely related to the effect of immunotherapy and prognosis.

Although the present study mostly achieved our initial vision, some shortcomings remain. Due to insufficient clinically relevant information in some of the GEO datasets we collected, we could not analyze the screened diagnostic genes and immune cell infiltration characteristics with clinicopathological characteristics in depth. In the same way, inadequate follow-up information prevented us from exploring and cross-validating immune cell infiltration characteristics with prognosis. Second, this study was analyzed exclusively based on public databases, lacking the validation of our relevant data, and may be subject to some bias. Lastly, the results are cross-validated and partly backed up by evidence from experiments, but they are still bioinformatic analyses that need to be confirmed by more experiments. This study was only done at the level of transcripts. GC diagnostic markers and immune cell infiltration characteristics would be easier to find with a full multi-omics and multi-dimensional analysis.

## 5 Conclusion

In summary, we screened eight candidate GC diagnostic genes using bioinformatics analysis with 2 ML algorithms and finally identified six diagnostic genes after cross-validation using AUC and other indicators. We also analyzed the infiltration of immune cells in GC and performed a correlation analysis between diagnostic genes and immune cell infiltration characteristics. The screened diagnostic genes were closely related to immune cell infiltration and had a definite prognostic value.

## Data Availability

The original contributions presented in the study are included in the article/Supplementary Material; further inquiries can be directed to the corresponding author.

## Supplementary material

Supplementary Figure S1 Merging of GEO datasets with the removal of batch effects **(A)** Before merging, the intersection of each GEO dataset. **(B)** The box plot indicates that the sample distribution of each dataset before removing the batch effect is different, suggesting a batch effect. **(C)** After removing the batch effect, the data distribution tends to be consistent among the data sets, and the medians are basically located on a horizontal line. **(D)** The density plot shows that the sample distribution of each data set before removing the batch effect is different, suggesting a batch effect. **(E)** After removing the batch effect, the data distribution of each data set tends to be consistent, with a similar mean and variance.

Supplementary Figure S2 Expression of candidate diagnostic genes in the validation set A **(A–H)** Scatter plots show candidate diagnostic genes' expression between tumor and normal groups in the validation set A. Red indicates the tumor group, and blue indicates the normal group. *p* < 0.05 shows a significant difference.

Supplementary Figure S3 Expression of candidate diagnostic genes in the validation set B **(A–H)** Scatter plots show candidate diagnostic genes' expression between tumor and normal groups in the validation set B. Red indicates the tumor group, and blue indicates the normal group. *p* < 0.05 shows a significant difference.

Supplementary Figure S4 ROC curves in the validation set A. **(A–H)** The ROC curves for the eight candidate diagnostic genes in the validation set A are shown in the figure. The horizontal coordinate is the false positive rate, presented as 1-specificity, and the vertical coordinate is the true positive rate, presented as sensitivity.

Supplementary Figure S5 ROC curves in the validation set B. **(A–H)** The ROC curves for the eight candidate diagnostic genes in the validation set B are shown in the figure. The horizontal coordinate is the false positive rate, presented as 1-specificity, and the vertical coordinate is the true positive rate, presented as sensitivity.

Supplementary Figure S6 Correlation of diagnostic genes with infiltrating immune cells **(A–F)** Demonstrate correlation between diagnostic genes and infiltrating immune cells, |R| ≥ 0.3 and *p* < 0.05, considered to be correlated.
