# Supplementary figures and images for: Identification of the diagnostic genes and immune cell infiltration characteristics of gastric cancer using bioinformatics analysis and machine learning
Source: Front Genet. 2023 Jan 4;13:1067524. doi: 10.3389/fgene.2022.1067524 (PMC9845288; doi:10.3389/fgene.2022.1067524)

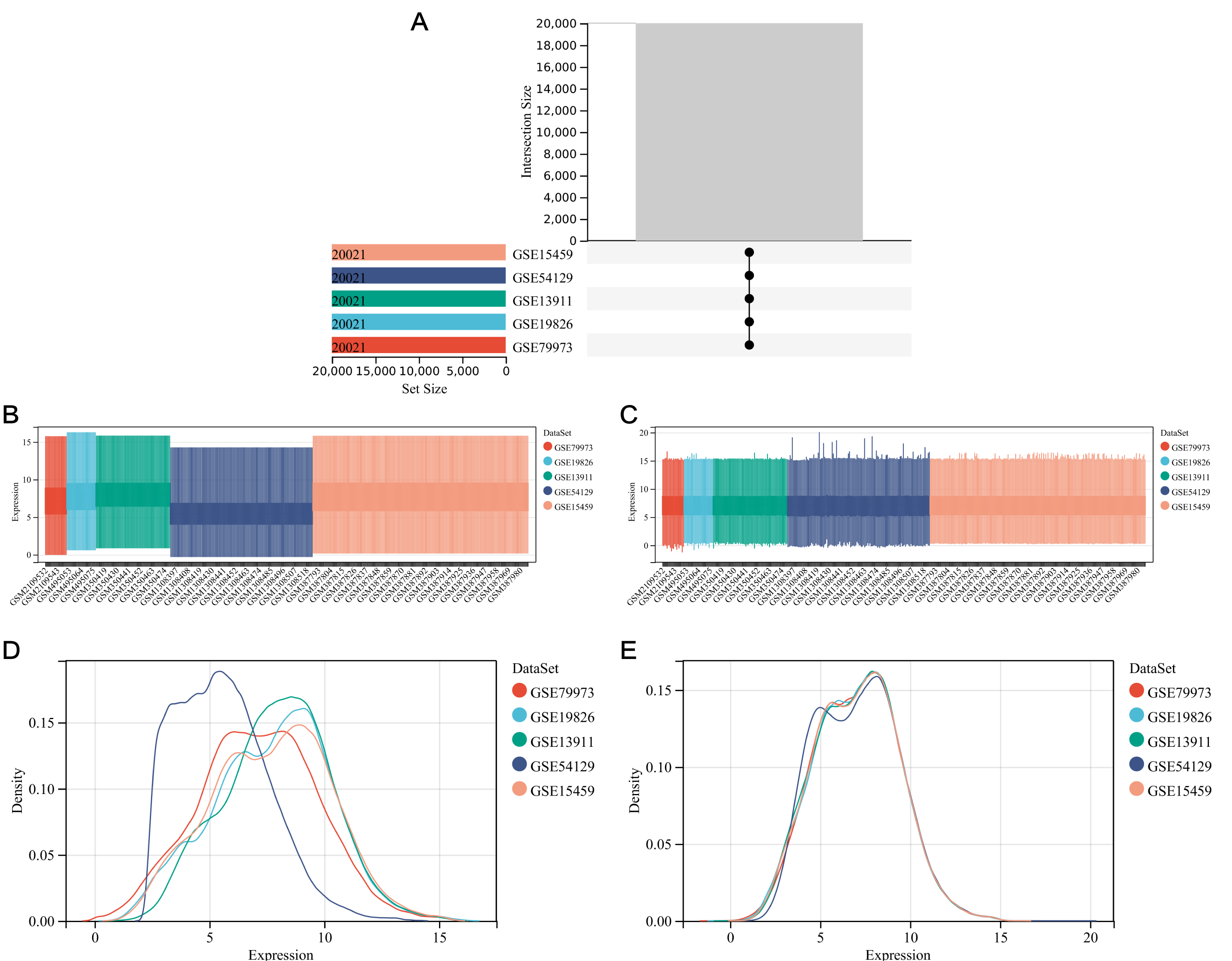

Supplement: Supplementary file 1 [file DataSheet1.ZIP › Supplementary Figure 1-6/Supplementary Figure 1.tif]

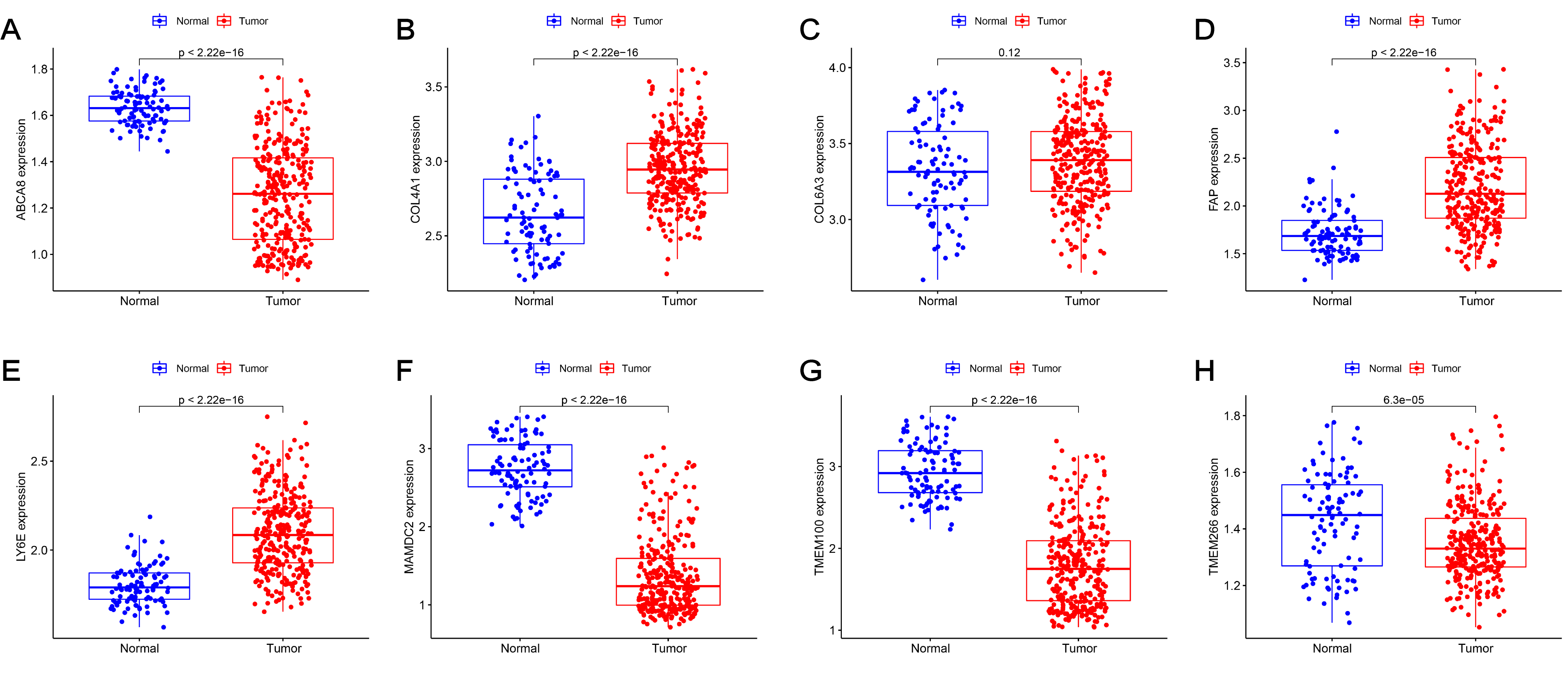

Supplement: Supplementary file 1 [file DataSheet1.ZIP › Supplementary Figure 1-6/Supplementary Figure 2.tif]

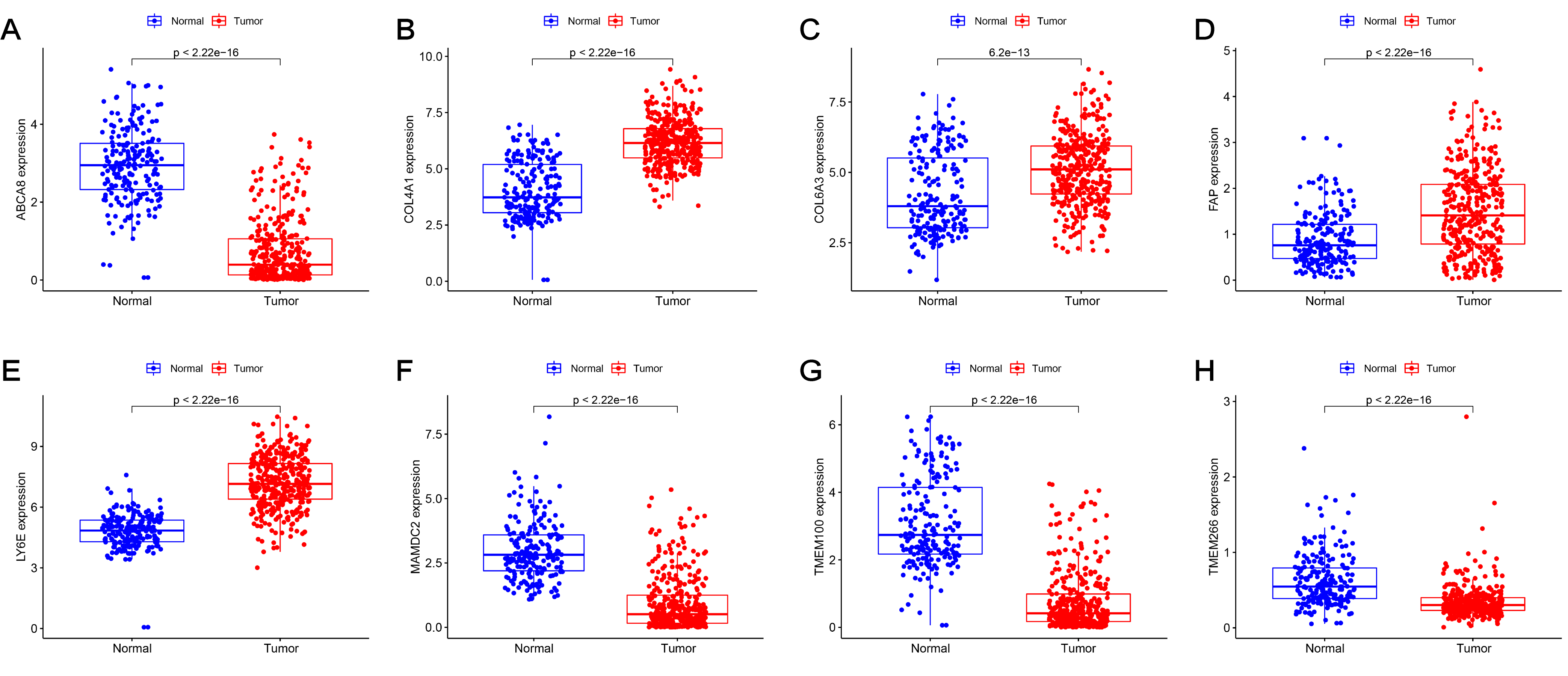

Supplement: Supplementary file 1 [file DataSheet1.ZIP › Supplementary Figure 1-6/Supplementary Figure 3.tif]

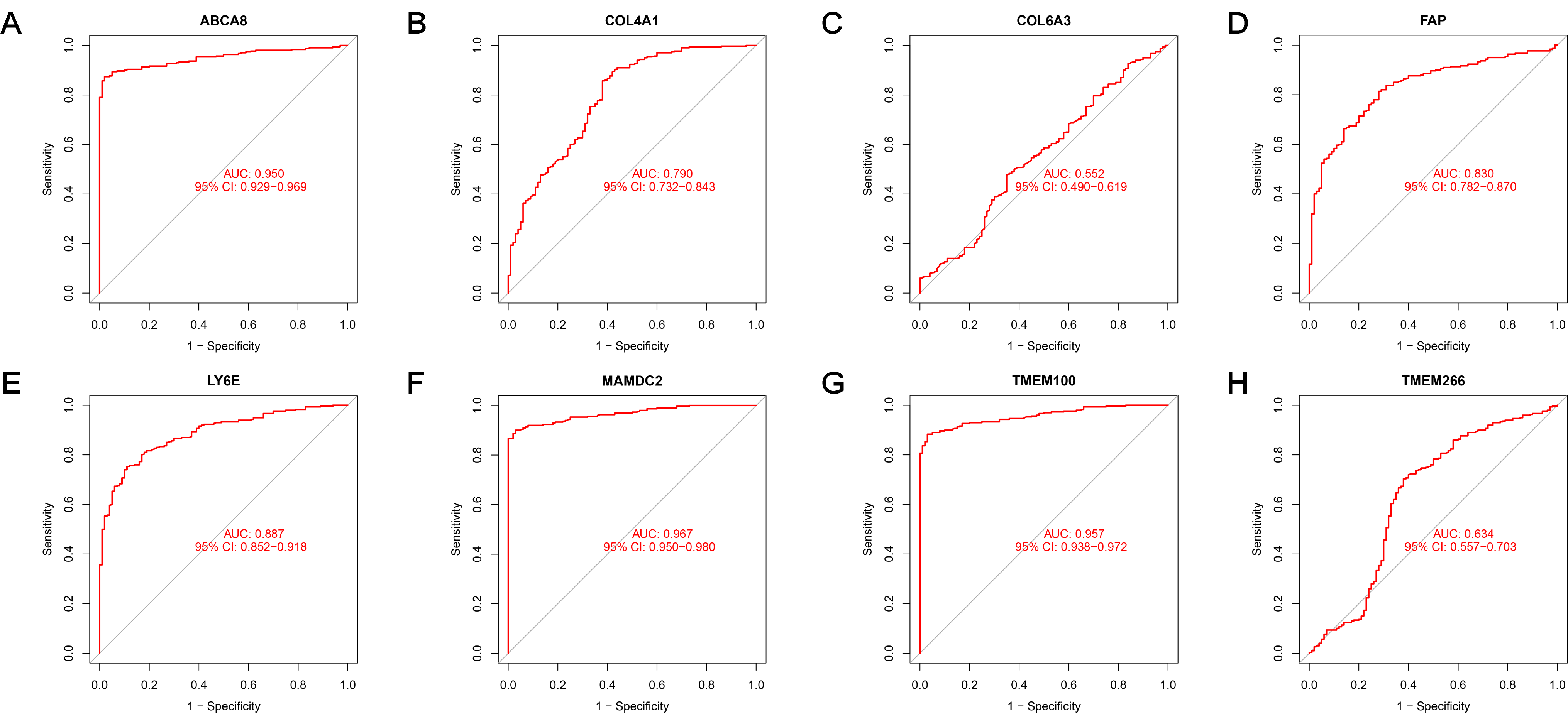

Supplement: Supplementary file 1 [file DataSheet1.ZIP › Supplementary Figure 1-6/Supplementary Figure 4.tif]

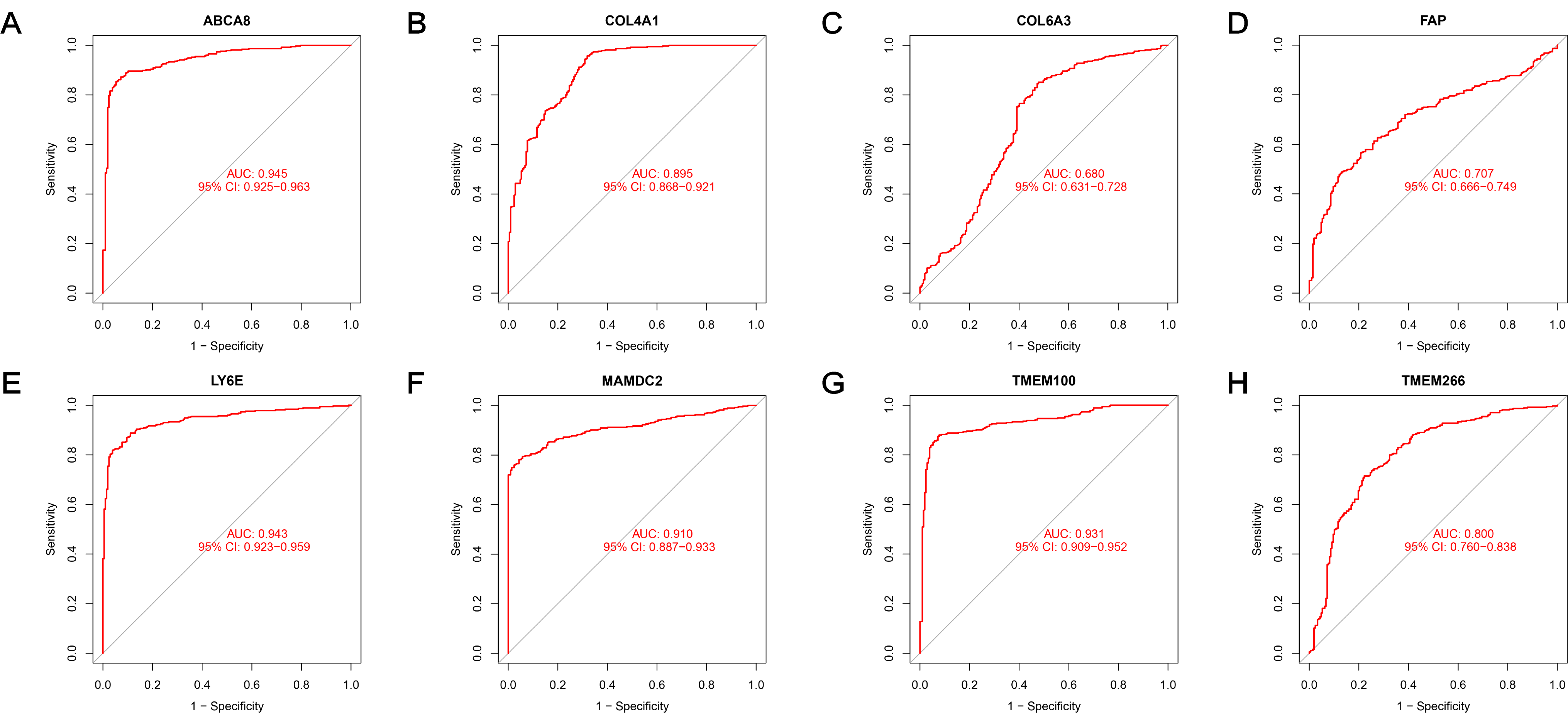

Supplement: Supplementary file 1 [file DataSheet1.ZIP › Supplementary Figure 1-6/Supplementary Figure 5.tif]

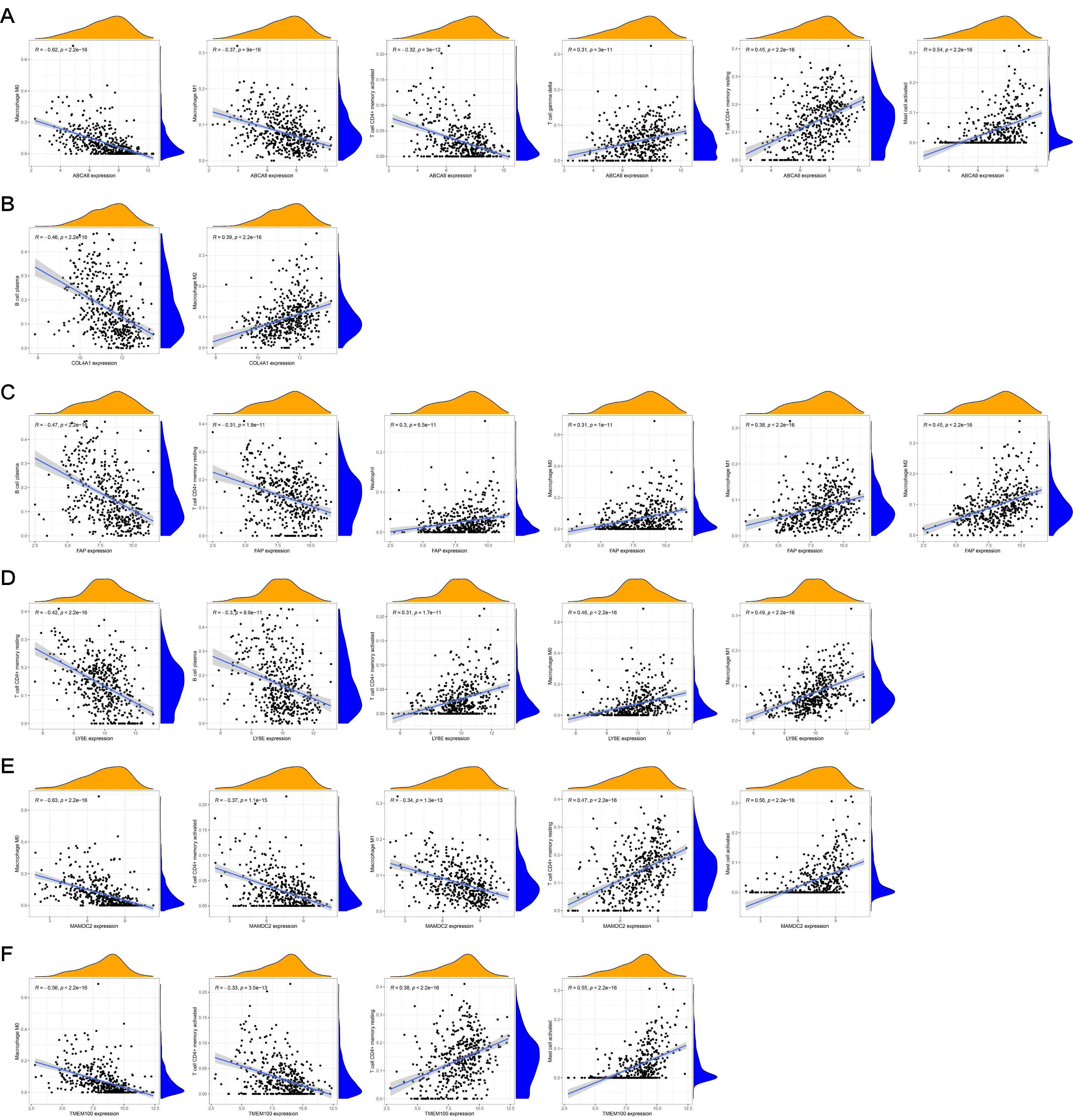

Supplement: Supplementary file 1 [file DataSheet1.ZIP › Supplementary Figure 1-6/Supplementary Figure 6.tif]
